# Geroderma Osteodysplastica in Two Patients: Clinical, Genetic, and Management Insights

**DOI:** 10.14740/jmc5343

**Published:** 2026-07-01

**Authors:** Waleed Almutairi, Ahmed Alibrahim, Faisal Alrashed, Obaid Alfuraydi

**Affiliations:** a Division of Endocrinology and Metabolism, Department of Medicine, King Abdulaziz Medical City, Riyadh, Saudi Arabia; b College of Medicine, King Saud bin Abdulaziz University for Health Sciences, Riyadh, Saudi Arabia

**Keywords:** Geroderma osteodysplastica, Connective tissue disorder, Management

## Abstract

Geroderma osteodysplastica (GO) is a rare autosomal recessive connective tissue disorder caused by pathogenic variants in the *GORAB* gene, characterized by premature skin ageing, joint laxity, and osteoporosis leading to recurrent fragility fractures. We described two adult Saudi patients with genetically confirmed GO. The first case involved a 34-year-old male with incidental vertebral fractures, congenital hip dislocation, and low bone mineral density (BMD) in the absence of secondary causes. The second case was a 35-year-old female with longstanding skeletal fragility, scoliosis, and prior hip fixation, also showing markedly reduced BMD. Both patients were born to consanguineous parents and had unremarkable hormonal and metabolic evaluations. Genetic testing confirmed the presence of homozygous pathogenic *GORAB* variants. Management included bone-directed therapy—romosozumab in the male patient and zoledronic acid in the female—along with calcium and vitamin D supplementation. These cases expand the phenotypic and therapeutic spectrum of GO and highlight the importance of considering rare genetic causes of osteoporosis in young adults, especially within consanguineous populations. Early recognition, genetic confirmation, and multidisciplinary management are crucial for optimizing outcomes and improving quality of life in affected individuals.

## Introduction

Geroderma osteodysplastica (GO) is a rare autosomal recessive connective tissue disorder characterized by premature ageing features and skeletal fragility [1]. The condition appears to be more prevalent in the Middle East, particularly in Oman, where the largest series of cases have been reported [2]. GO was first described by Bamatter et al in 1950, who reported five affected individuals from a Swiss family [1].

The clinical phenotype of GO is distinctive and includes a prematurely aged, sagging face with drooping eyelids, jowly appearance, and malar hypoplasia. Other common findings are lax and wrinkled atrophic skin—especially on the extremities—an arm span that exceeds height, kyphoscoliosis, joint hyperextensibility (particularly in the hands and feet), osteoporosis, hypotonia, and preserved intelligence [3–6].

GO is caused by pathogenic variants in the *GORAB* gene located on chromosome 1q24.2 [7]. The *GORAB* gene encodes a Golgi-associated coiled-coil protein that interacts with RAB6, a key regulator of retrograde trafficking between the Golgi apparatus and endoplasmic reticulum [8]. This interaction is essential for maintaining Golgi structure and for proper collagen biosynthesis and trafficking. Loss-of-function mutations disrupt this process, leading to defective Golgi function and impaired collagen synthesis, which underlie the characteristic cutaneous and skeletal manifestations of the disease [9, 10].

In this report, we described two Saudi patients presenting with recurrent fractures and classical phenotypic features of GO. By sharing their clinical, radiological, and genetic findings, we aim to raise awareness of this rare disorder, emphasize its consideration in the differential diagnosis of unexplained fractures and support early recognition for optimized patient care.

## Case Reports

### Case 1

A 34-year-old Saudi male with a past medical history of depression and anxiety disorder was referred to the endocrine clinic after multiple thoracolumbar compression fractures were incidentally detected on a computed tomography (CT) scan of the abdomen and pelvis performed for renal stones (Fig. 1).

**Figure 1:**
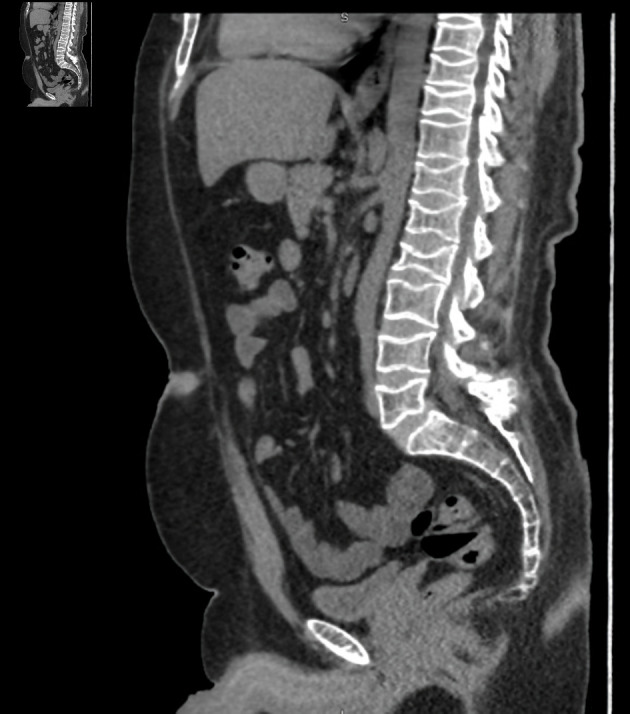
CT scan of abdomen and pelvis showing generalized decreased bone density with multiple compression fractures of thoracolumbar spine. CT: computed tomography.

On further history, he reported bilateral congenital hip dislocation (surgically corrected on the right side) and a previous left ankle fracture sustained after a simple sprain. He was born to consanguineous parents and has eight siblings, none of whom have a history of recurrent fractures or bone disorders. He denied smoking, alcohol consumption, or the use of regular medications or supplements, including corticosteroids.

On physical examination, he appeared of average build with a body mass index (BMI) of 31.6 kg/m^2^ (height 154 cm, weight 75 kg). Notable dysmorphic features included low-set ears and thin, wrinkled skin over the face and hands. Secondary sexual characteristics were appropriate for age; full pubertal development with Tanner stage V genitalia (testicular volume approximately 25 mL) and Tanner stage V pubic hair.

Laboratory evaluation, including a complete hormonal profile, is shown in Table 1. Initial laboratory evaluation showed a mildly reduced serum phosphate level and a low luteinizing hormone (LH) concentration. Given these findings, further evaluation was performed. Repeat biochemical testing demonstrated normalization of serum phosphate, LH, and follicle-stimulating hormone (FSH) levels. Total testosterone remained within the normal range, and the patient exhibited normal virilization and age-appropriate secondary sexual characteristics. Pituitary magnetic resonance imaging (MRI) was unremarkable, with no evidence of structural hypothalamic–pituitary abnormalities. No biochemical or clinical evidence of phosphate-wasting disorders, hypogonadotropic hypogonadism, or other metabolic bone diseases was identified. These isolated abnormalities were therefore considered transient and not clinically relevant to the patient's underlying skeletal disorder.

**Table 1:** Laboratory Evaluation of Case 1

Exam	Result	Reference range
Hb	164	130–170 g/L
WBC	9.31	4.0–11.0 × 10^9^/L
Platelet	252	150–400 × 10^9^/L
HbA1c	5.1%	≤ 5.6%
Creatinine	59	60–110 µmol/L
eGFR	145	> 90 mL/min/1.73 m^2^
Adjusted calcium	2.34	2.15–2.55 mmol/L
Phosphorus	0.66	0.8–1.5 mmol/L
Magnesium	0.84	0.7–1.0 mmol/L
Uric acid	366	210–420 µmol/L
TV urine	2,500	1,000–2,500/24 h
U24 calcium	4.58	2.5–7.5 mmol/24 h
U24 creatinine	13.5	9–18 mmol/24 h
Total bilirubin	11.4	5–21 µmol/L
Alkaline phosphatase	77	40–129 U/L
Total protein	79	60–80 g/L
Albumin	52	35–50 g/L
ALT	17	≤ 41 U/L
AST	20	≤ 40 U/L
Testosterone total	21.09	8.0–30.0 nmol/L
LH	0.98	1.7–8.6 IU/L
FSH	2.10	1.5–12.4 IU/L
Prolactin	144.93	86–324 mIU/L
TSH	1.44	0.4–4.0 mIU/L
FT4	12.15	10–23 pmol/L
PTH	3.110	1.6–6.9 pmol/L
LDL	3.92	< 2.6 mmol/L
HDL	1.26	≥ 1.0 mmol/L
Total cholesterol	5.40	< 5.2 mmol/L
Triglycerides	0.67	< 1.7 mmol/L
Albumin E	43.1	35–50 g/L
Alpha 2	6.3	4.8–10.5%
Beta 1	4.7	4.7–7.2%
Beta 2	4.8	3.2–6.5%
Gamma	10.3	9–18%
Pro El Interp	Normal	Normal pattern
Alpha 1	2.8	1.4–3.0%
M spike g/L	0	Absent
M spike %	0	Absent
25-OH Vit D	80.7	50–125 nmol/L
Tissue transglutaminase IgA antibody	0.89	< 10 U/mL (negative)

Hb: hemoglobin; WBC: white blood cell; HbA1c: hemoglobin A1c; eGFR: estimated glomerular filtration rate; TV urine: 24-h urine total volume; U24 calcium: 24-h urinary calcium; U24 creatinine: 24-h urinary creatinine; ALT: alanine aminotransferase; AST: aspartate aminotransferase; LH: luteinizing hormone; FSH: follicle-stimulating hormone; TSH: thyroid-stimulating hormone; FT4: free thyroxine; PTH: parathyroid hormone; LDL: low-density lipoprotein cholesterol; HDL: high-density lipoprotein cholesterol; Alpha 1: alpha-1 globulin; Alpha 2: alpha-2 globulin; Beta 1: beta-1 globulin; Beta 2: beta-2 globulin; Gamma: gamma globulin; Pro El Interp: protein electrophoresis interpretation; M spike: monoclonal protein spike; VIT D 25 OH: 25-hydroxyvitamin D.

No M-protein was detected on protein electrophoresis as well. Dual-energy X-ray absorptiometry (DEXA) demonstrated low bone mineral density (BMD) for age, with right proximal femur Z-score of −2.7, and right femoral neck Z-score of −1.8 (Fig. 2) and a lumbar spine Z-score of −3.0 (Fig. 3).

**Figure 2:**
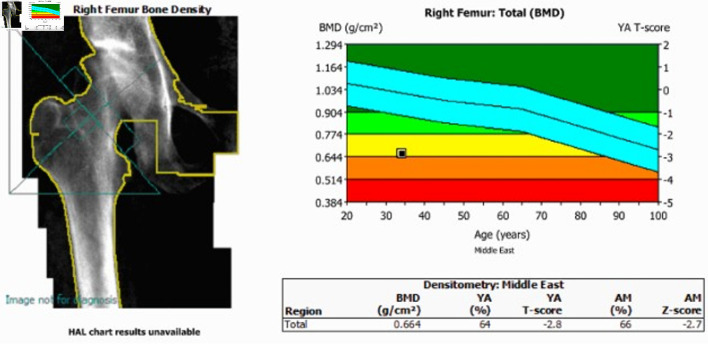
Femur bone mass density. DEXA scan showing a right proximal femur Z-score of −2.7, and a right femoral neck Z-score of −2.8. BMD: bone mineral density; DEXA: dual-energy X-ray absorptiometry.

**Figure 3:**
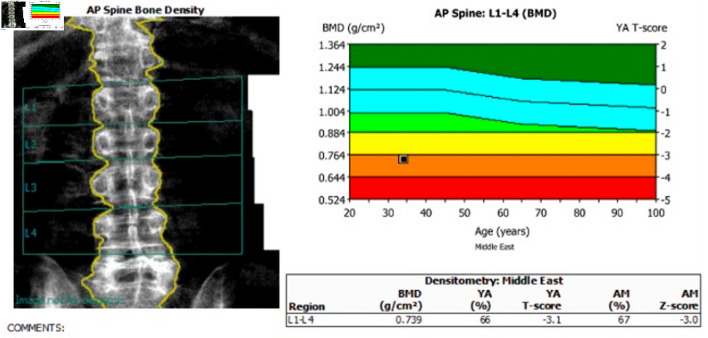
Spine bone mass density. DEXA scan showing a lumbar spine Z-score of −3.0. DEXA: dual-energy X-ray absorptiometry.

Genetic testing confirmed a biallelic pathogenic variant in *GORAB*, establishing the diagnosis of autosomal recessive GO. Molecular genetic testing identified a homozygous pathogenic frameshift variant in *GORAB*, NM_152281.2.306dup (p.(Pro103Thrfs*20)). The variant was classified as pathogenic (class 1) according to ACMG-based laboratory criteria. The variant has previously been reported as disease-causing for GO and is predicted to result in premature protein truncation.

Given the severity of osteoporosis in this patient characterized by multiple fragility fractures, markedly reduced BMD, progressive skeletal complications, and limited evidence-based therapeutic alternatives, treatment with romosozumab was considered despite its off-label status. Before treatment initiation, a comprehensive cardiovascular assessment was performed, which revealed no history of myocardial infarction, stroke, or other major cardiovascular disease. The potential benefits, risks, and uncertainties associated with romosozumab therapy, including its black-box warning regarding cardiovascular events and the absence of published evidence in GO, were thoroughly discussed with the patient. The off-label nature of treatment was explicitly explained, and alternative management options were reviewed. Following a shared decision-making process, the patient provided informed consent to proceed with therapy.

The patient was initiated on monthly romosozumab injections for a duration of 1 year, in conjunction with cholecalciferol 50,000 IU once monthly and calcium carbonate 600 mg daily. He was also referred to the genetic counseling team for family education and screening. After receiving eight doses, he sustained a left ankle fracture following a rolling injury that required surgical fixation at another hospital. During subsequent clinic follow-up, he reported missing three scheduled doses. After careful assessment, the fracture was considered traumatic rather than a fragility fracture; therefore, completion of the remaining four doses was recommended to achieve a total of 12 doses of romosozumab. Repeat DXA demonstrated stable BMD. Following completion of romosozumab therapy, the patient elected to continue osteoporosis management with zoledronic acid after counseling regarding available treatment options. Bone turnover markers were not available at our institution; therefore, treatment response was assessed using clinical outcomes and serial DXA measurements. Serum calcium, vitamin D, and renal function remained within acceptable ranges throughout follow-up, and no treatment-related adverse events were observed.

### Case 2

A 35-year-old Saudi female (divorced, nulliparous) with a past medical history of stable localized scleroderma managed by the dermatology service, sleeve gastrectomy (2010), and depressive disorder (since 2017) under regular psychiatric follow-up, was referred to the endocrinology clinic for evaluation of recurrent fragility fractures involving the spine and lower extremities.

On further history, the patient reported a remote right hip fracture more than 20 years ago, following minor trauma (fall from a standing height), which required surgical fixation. Her menstrual history was normal, with regular cycles and no evidence of amenorrhea or hormonal imbalance. She was born to consanguineous parents. There was no family history of recurrent fractures, renal stones, or similar complaints. Her current medications included methotrexate 15 mg weekly, folic acid 5 mg, venlafaxine extended-release 225 mg, and lamotrigine 200 mg daily. She was not taking any vitamin supplements. She also reported a remote history of intravenous steroid use approximately 5 years earlier for a scleroderma flare, with no regular steroid use since then.

On examination, she was obese (BMI 43 kg/m^2^) and wheelchair-bound, with significant limitations in her daily activities. Back examination revealed scoliosis and mild vertebral tenderness. The gynecological examination was unremarkable.

A comprehensive laboratory evaluation, including a hormonal profile, is presented in Table 2. The elevated parathyroid hormone (PTH) level was interpreted in the context of vitamin D deficiency and was considered most consistent with secondary hyperparathyroidism. The patient underwent assessment of calcium, phosphate, vitamin D status, renal function, and other biochemical parameters relevant to metabolic bone disease. Nutritional deficiencies and the potential impact of prior bariatric surgery were considered; however, these factors alone were not felt to explain the severity of osteoporosis, the characteristic skeletal manifestations adequately, or the confirmed pathogenic *GORAB* mutation. Similarly, prior glucocorticoid exposure and reduced mobility may have contributed to bone loss but were considered additional risk factors rather than the primary etiology of the patient’s skeletal disease. Imaging studies were obtained, including a hip radiograph (Fig. 4) and a BMD assessment. A scoliosis survey (Fig. 5) confirmed spinal deformity and multilevel disc disease. Hip radiography demonstrated a right-sided intramedullary nail abutting the iliac bone, with no evidence of new pelvic fractures or dislocations. DEXA revealed markedly reduced BMD, with a lumbar spine Z-score of –1.9, right proximal femur Z-score of –0.6, and right femoral neck Z-score of –2.9, consistent with BMD below the expected range for age.

**Table 2:** Laboratory Evaluation of Case 2

Exam	Result	Reference range
WBC	6.10	4.0–11.0 × 10^9^/L
Hb	118	115–155 g/L
Platelet	260	150–400 × 10^9^/L
Ferritin	9.4	15–150 µg/L
HbA1C	5.5	≤ 5.6% normal
Creatinine	43	45–90 µmol/L
BUN	4.3	2.5–7.1 mmol/L
eGFR	153	> 90 mL/min/1.73 m^2^
Sodium	138	135–145 mmol/L
Potassium	3.8	3.5–5.0 mmol/L
Adjusted calcium	2.21	2.15–2.55 mmol/L
Phosphorus	1.28	0.8–1.5 mmol/L
Magnesium	0.86	0.7–1.0 mmol/L
ALT	23	≤ 33 U/L
AST	19	≤ 32 U/L
Total bilirubin	6.3	5–21 µmol/L
Alkaline phosphatase	70	35–104 U/L
Total protein	63	60–80 g/L
FSH	4.34	3.5–12.5 IU/L (follicular), 1.7–7.7 IU/L (luteal)
LH	6.91	2.4–12.6 IU/L (follicular), 0.5–16.9 IU/L (luteal)
Testosterone total	2.14	0.3–2.4 nmol/L
TSH	1.56	0.4–4.0 mIU/L
Prolactin	300.9	102–496 mIU/L
Total 25-OH Vit D	64.0	50–125 nmol/L
PTH	11.49	1.6–6.9 pmol/L
LDL	3.49	< 3.0 mmol/L
HDL	1.19	> 1.0 mmol/L
Total cholesterol	5.05	< 5.2 mmol/L
Triglycerides	1.69	< 1.7 mmol/L

WBC: white blood cell; Hb: hemoglobin; HbA1c: hemoglobin A1c; BUN: blood urea nitrogen; eGFR: estimated glomerular filtration rate; ALT: alanine aminotransferase; AST: aspartate aminotransferase; LH: luteinizing hormone; FSH: follicle-stimulating hormone; TSH: thyroid-stimulating hormone; PTH: parathyroid hormone; LDL: low-density lipoprotein cholesterol; HDL: high-density lipoprotein cholesterol; 25-OH Vit D: 25-hydroxyvitamin D.

**Figure 4:**
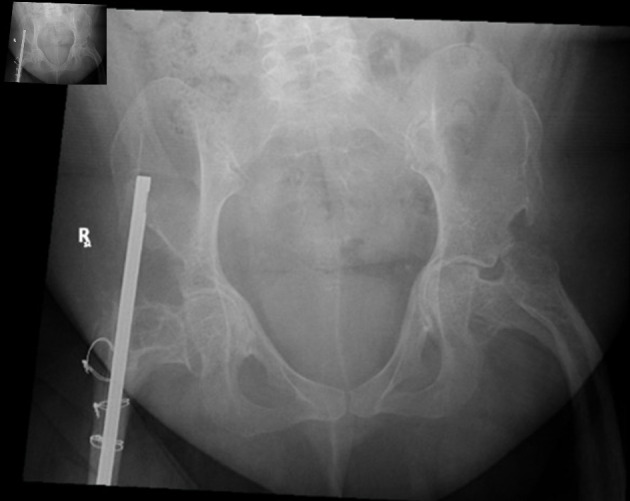
Hip X-ray. The right-sided intramedullary nail is abutting the right iliac wing with no signs of hardware loosening or metallic fracture. There are no fractures or dislocations noted in the pelvic bones.

**Figure 5:**
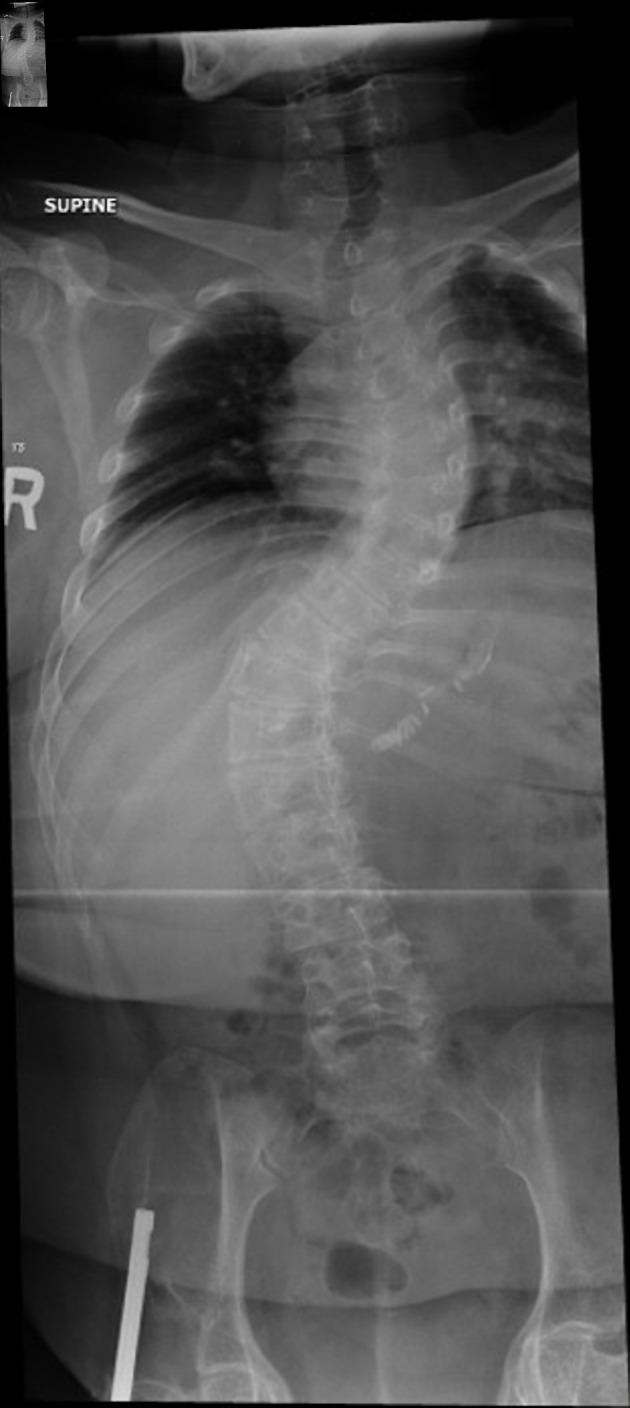
Scoliosis series X-ray. Scoliosis of the thoracolumbar spine is noted as reversed S-shape. The upper curvature has a convexity to the left side and affects the mid-thoracic spine. The lower convexity is affecting the thoracolumbar junction with convexity to the right side. Asymmetry of the vertebral body heights is also noted also at the described scoliotic curvatures.

Given these findings, genetic testing was pursued. Targeted Sanger sequencing identified a homozygous pathogenic variant in the *GORAB* gene, NM_152281.2.306dup (p.(Pro103Thrfs*20)), confirming the diagnosis of autosomal recessive GO. Thus, both patients carried an identical homozygous pathogenic frameshift variant in *GORAB*.

After discussion of management options (including anabolics), the patient elected to proceed with an annual zoledronic acid infusion, along with calcium and vitamin D supplementation, and was followed for 3 years. She declined referral to a genetic counseling team. During follow-up, no new fractures were reported. Follow-up DXA demonstrated approximately a 5% improvement in BMD at the hip. Based on this favorable response, continuation of zoledronic acid therapy for an additional 2 years was planned. Serum calcium, vitamin D, and renal function were monitored before and after treatment and remained stable. No significant adverse events related to zoledronic acid administration were observed. Comparison of case 1 and case 2 are presented in Table 3.

**Table 3:** Comparison of Both Cases

	Case 1	Case 2
Age	34 years old	35 years old
Gender	Male	Female
Major clinical features	Osteoporosis, multiple fragility fractures, congenital hip dislocation, and low BMD	Prior steroid exposure, obesity, wheelchair dependence, low ferritin, and elevated PTH, scleroderma, longstanding skeletal fragility, scoliosis, and prior hip fixation and low BMD
Fracture history	Thoracolumbar compression fractures, left ankle fracture	Right hip fracture
BMD values	Right proximal femur Z-score of −2.7, and right femoral neck Z-score of −1.8; a lumbar spine Z-score of −3.0	Lumbar spine Z-score of–1.9, right proximal femur Z-score –0.6, and right femoral neck Z-score –2.9
Genetic variant	*GORAB*, NM_152281.2.306dup (p.(Pro103Thrfs*20))	*GORAB*, NM_152281.2.306dup (p.(Pro103Thrfs*20))
Treatment	Romosozumab for 1 year, cholecalciferol 50,000 IU/month, calcium carbonate 600 mg/day	Annual zoledronic acid infusion, along with calcium and vitamin D supplementation
Follow-up and outcomes	Romosozumab was administered for 8 doses initially and later completed with an additional 4 doses; BMD remained stable, followed by treatment with zoledronic acid	No new fracture or adverse event report during 3-year follow-up, and 5% BMD improved

BMD: bone mineral density; PTH: parathyroid hormone.

## Discussion

GO, also known as osteodysplastic geroderma, is a rare autosomal recessive connective tissue disorder characterized by skin laxity, progeroid facies, joint hyperlaxity, and severe osteoporosis with recurrent fragility fractures [11]. To date, reports of GO from the Middle East remain scarce, and consists predominantly of phenotypic and genetic case reports from Saudi Arabia [2,12,13], Oman [14], United Arab Emirates [15], Kuwait [16], and Lebanon [1], highlighting the rarity of the disorder and the importance of genetic evaluation in populations with a high prevalence of consanguinity. The largest regional series was reported by Rajab et al [14], who described 22 affected individuals from 11 Omani families and highlighted the characteristic osteopenia, recurrent fractures, and premature aged appearance associated with the disorder. Despite the substantial skeletal burden reported in these patients, data regarding pharmacological management of osteoporosis in GO are extremely scarce. Our report expands the existing literature by describing two Saudi patients with GO treated with romosozumab and documenting their skeletal response. To our knowledge, this represents the first report from Saudi Arabia and among the first worldwide to provide detailed clinical outcomes following anabolic osteoporosis therapy in GO, thereby contributing valuable evidence toward the management of bone fragility in this rare genetic disorder.

Our first patient, a 34-year-old male, presented with incidental vertebral compression fractures, congenital hip dislocation, and skeletal fragility in the absence of overt endocrine abnormalities. The second patient, a 35-year-old female, had a more overt fracture history spanning decades, with concomitant comorbidities including scleroderma, and prior steroid exposure. Despite these differences, both shared key features: consanguineous parental background, absent family history of bone disease, and marked reductions in BMD disproportionate to traditional risk factors.

The skin and connective tissue findings—such as wrinkled or lax skin over the hands and face, progeroid facial features, and joint hyperlaxity—were subtle but present, especially in the male patient. These findings are consistent with prior descriptions of GO, which emphasize lax and prematurely aged skin, underdeveloped facial bones, and joint hypermobility. In previous reports [6], features such as radiographic peculiarities have been described as characteristic, though these are variably present. In our cases, the radiographic hallmarks centered on low BMD and vertebral fragility rather than specific epiphyseal anomalies.

Each of the above factors in both cases have been reported to adversely affect bone health through mechanisms including altered bone remodeling, reduced nutrient absorption, medication-related effects on bone metabolism, and increased risk of falls. Therefore, the patient’s low BMD and fracture history are likely multifactorial rather than attributable solely to GO. However, the presence of a confirmed connective tissue disorder known to be associated with osteoporosis and fragility fractures suggests that GO remains a significant contributor to the observed skeletal phenotype. Given the rarity of the condition and the coexistence of multiple acquired risk factors, the relative contribution of each factor cannot be precisely quantified, highlighting the complexity of assessing bone disease in patients with syndromic osteoporosis. This diagnostic conclusion underscores the necessity for comprehensive clinical assessments to differentiate primary genetic osteoporosis from secondary causes in the context of rare connective tissue disorders [17].

GO is extremely rare and often underrecognized, many patients suffer diagnostic delay, sometimes spanning decades [1]. In our series, the index male was diagnosed only after imaging for another indication, while the female’s long fracture history had not triggered a rare bone workup until late. These cases reinforce the need for heightened suspicion when clinicians face unexplained osteoporosis or fragility fractures in younger patients, especially in populations where consanguinity is common.

Genetic testing in both cases confirmed biallelic pathogenic variants in the *GORAB* gene (also known as *SCYL1BP1*), thereby clinching the diagnosis of autosomal recessive GO. The GORAB protein is localized to the Golgi apparatus and is implicated in vesicle trafficking, glycosylation and extracellular matrix homeostasis [8]. Animal and cellular models have shown that loss of GORAB leads to impaired proteoglycan glycosylation, disorganized collagen matrices, and increased transforming growth factor (TGF)-β activation, resulting in defective osteoblast differentiation, cortical thinning, and propensity to fracture [18]. Thus, GO might be conceptualized as a congenital disorder of glycosylation with downstream skeletal consequences.

Because there is no curative therapy for GO, management is largely supportive and aimed at reducing fracture risk, preserving mobility, and optimizing bone health [19]. Prior case reports have employed bisphosphonates (e.g., pamidronate, zoledronic acid) with variable success, often resulting in BMD stabilization or modest improvement and a reduction in fracture incidence. For example, an earlier Saudi case treated with pamidronate showed improvement in BMD over time [6]. In another recent GO report, bisphosphonate therapy decreased fractures [1]. Given the rarity of GO and the limited evidence available to guide treatment, ongoing pharmacovigilance and post-marketing safety surveillance are important to better characterize the long-term safety and effectiveness of therapeutic interventions in affected patients. Real-world safety data have increasingly contributed to clinical decision-making in rare disorders, as demonstrated by recent pharmacovigilance analyses of therapies used in other rare genetic diseases [20].

In case 1, we initiated romosozumab, a sclerostin inhibitor with dual anabolic and antiresorptive effects, administered monthly for 1 year in combination with calcium and high-dose vitamin D, followed by antiresorptive therapy to preserve gains in bone density. This strategy represents a more aggressive, contemporary approach to managing severe osteoporosis in relatively young adults, although specific evidence in GO is lacking. The therapeutic rationale was to stimulate bone formation prior to antiresorptive therapy. The decision to use romosozumab was individualized based on the patient’s severe fracture burden, ongoing skeletal deterioration, and the need for an anabolic treatment approach in the setting of limited available therapeutic options. In case 2, we employed a conventional regimen consisting of zoledronic acid infusion (5 mg) with calcium and vitamin D supplementation, which remains a practical and widely accessible treatment option in many clinical settings.

It is too early to draw firm conclusions about superiority of one regimen over another in GO. However, these two contrasting therapeutic choices in genetically confirmed cases provide a useful way for future observational follow-up. Close monitoring of BMD, bone turnover markers, fracture incidence, and safety (e.g., hypocalcemia, renal function) is essential.

Beyond bone-targeted therapy, a multidisciplinary approach is indispensable. Patients should undergo physical rehabilitation, fall-prevention strategies, orthopedic management when fractures or deformities occur, nutritional optimization, and, in appropriate cases, genetic counseling and family screening. Although the female patient declined genetic referral, we emphasize the importance of such counseling for both recurrence risk and potential supportive care for relatives.

Our experience with these cases highlights several important lessons and challenges. Clinicians should maintain a high index of suspicion for rare skeletal disorders like GO when encountering unexplained fragility fractures in young individuals, particularly in populations with consanguinity. Subtle physical findings—such as wrinkled skin, facial dysmorphism, and joint laxity—can provide critical diagnostic clues. Confirming a genetic diagnosis not only clarifies management but also shifts focus on long-term skeletal protection and family counseling. Because evidence for GO treatment remains limited, cautious therapeutic exploration with newer osteoporosis agents may be justified within research settings. Establishing registries and collecting longitudinal data are crucial for better understanding genotype–phenotype relationships and guiding care. Nonetheless, the present report is limited by short follow-up, limited bone analyses, and confounding comorbidities, which together temper the strength of the conclusions drawn. Furthermore, parental testing and segregation analysis were unavailable for either patient, limiting the ability to characterize the inheritance pattern in this family cohort definitively.

Future research on GO should focus on building collaborative, prospective studies to clarify its optimal management. Establishing comprehensive registries that include detailed clinical, radiologic, and genetic information will be key to understanding disease variability. Investigating bone turnover markers and histomorphometric profiles could shed light on the underlying remodeling processes. Small-scale trials exploring newer anabolic agents such as teriparatide or romosozumab may help assess their potential benefits and safety in GO patients. In parallel, functional studies examining the molecular effects of *GORAB* mutations could reveal novel therapeutic targets, such as pathways involving TGF-β signaling or proteoglycan synthesis. Finally, proactive genetic counseling and family screening can aid in early detection and preventive strategies for at-risk individuals.

### Conclusions

GO is a rare autosomal recessive disorder characterized by premature skin ageing, connective tissue laxity, and early-onset osteoporosis, leading to recurrent fractures. Our two Saudi patients illustrate the diagnostic challenges and clinical variability of this condition. Recognition of GO in adults with unexplained fragility fractures, particularly in consanguineous populations, is essential for timely genetic confirmation and tailored management. Although no curative therapy exists, antiresorptive and anabolic treatments may improve bone health. Multidisciplinary care and genetic counseling remain key to optimizing outcomes and expanding understanding of this rare disease.

### Learning points

Diagnostic awareness: GO should be considered in patients presenting with early-onset osteoporosis combined with distinctive skin laxity and progeroid facial features, particularly in consanguineous families.

Geographic relevance: High rates of consanguinity in Middle Eastern populations increases the likelihood of autosomal recessive disorders like GO; therefore, enhanced awareness and surveillance are warranted in Saudi Arabia and other Gulf region countries.

Genotypic heterogeneity: *GORAB* mutations display significant diversity, and novel variants continue to expand the mutational spectrum, requiring individualized genetic counseling.

Phenotypic variability: Atypical presentations, such as tall stature instead of short stature, may delay diagnosis; therefore, clinical vigilance is essential.

Therapeutic implications: Early initiation of bisphosphonate therapy and multidisciplinary care can significantly improve quality of life and reduce morbidity in GO patients.

## Data Availability

The authors declare that data supporting the findings of this study are available within the article.
